# A case of familial partial lipodystrophy type 2 masquerading as Cushing syndrome: Explaining an atypical phenotype by whole-exome sequencing

**DOI:** 10.20945/2359-4292-2024-0293

**Published:** 2025-03-11

**Authors:** Enid Perez-Dionisio, Silvia Hinojosa-Alvarez, Rocio Alejandra Chavez-Santoscoy, Regina de Miguel-Ibañez, Manuel Garcia-Saenz, Daniel Marrero-Rodriguez, Keiko Taniguchi-Ponciano, Jesus Henandez-Perez, Moises Mercado, Claudia Ramirez-Renteria, Ernesto Sosa-Eroza, Etual Espinosa-Cardenas

**Affiliations:** 1 Servicio de Endocrinología, UMAE Hospital de Especialidades, Centro Médico Nacional Siglo XXI, Instituto Mexicano del Seguro Social, Ciudad de México, México; 2 Unidad de Investigación Médica en Enfermedades Endocrinas, UMAE Hospital de Especialidades, Centro Médico Nacional Siglo XXI, Instituto Mexicano del Seguro Social, Ciudad de México, México; 3 Escuela de Ingeniería y Ciencias, Instituto Tecnológico de Monterrey, Monterrey, México

## Abstract

Familial partial lipodystrophy type 2 is a rare disease, particularly when it is caused by nonclassical gene variants. A high index of suspicion is essential for a timely diagnosis. We present the case of a 32-year-old woman, referred to evaluation of a possible Cushing syndrome, which was clinically and biochemically ruled out. Yet, due to the finding of a rather abnormal fat distribution during physical examination, the diagnosis of lipodystrophy was cogitated. Whole-exome sequencing revealed a missense variant of exon 11 R582H of the gene encoding Laminin A (rs57830985,c.1745G>A, p.Arg582His). The patient presented some clinical and biochemical characteristics discordant with those previously reported in patients harboring other classical variants of this gene.

## INTRODUCTION

Lipodystrophies are a group of heterogeneous, low prevalence diseases (one case per million) (^1^), characterized by total or partial loss of adipose tissue, which can be congenital or acquired. Although their most constant feature is adipose tissue hypotrophy, some subtypes present with a rather abnormal distribution of fat, which can accumulate in certain organs (^1^,^2^). Some lipodystrophies are accompanied by metabolic alterations, namely: insulin resistance; type 2 diabetes (T2D); hypertriglyceridemia; non-alcoholic fatty liver disease (NAFLD); hypoalphalipoproteinemia, and polycystic ovary syndrome (^3^).

Different subtypes of lipodystrophy include generalized congenital lipodystrophy, acquired generalized lipodystrophy, familial (congenital) partial lipodystrophy, and acquired partial lipodystrophy. Dunnigan’s syndrome is the most common type of familial partial lipodystrophies (FPLD2), holding an autosomal dominant inheritance, although a more severe phenotype has been described in patients harboring homozygous pathogenic variants (^1^-^3^).

FPLD2 is caused by a missense pathogenic variant of the *LMNA* gene. This gene, located at 1q21-22 and consisting of 12 exons, encodes laminin A/C (LMNA/C), which along with laminin B, forms a protein network found below the inner nuclear membrane. These proteins are crucial for the preservation of nuclear membrane mechanical stability, anchoring proteins in the nuclear pore complex, and organizing chromatin; they also have a fundamental role in the regulation of DNA replication and transcription (^4^). So-called laminopathies are a group of diseases caused by various pathogenic variants of this gene. The most common or “typical” FPLD2 pathogenic variant is found in exon 8, specifically in codon R482, while all other pathogenic variants are known as atypical (^5^). The classical phenotype usually occurs in post-pubertal women; it is characterized by the loss of adipose tissue in the legs, arms, buttocks, and trunk and an accumulation of fat in the neck, face, dorsocervical region, labia majora, and visceral area, frequently resulting in a cushingoid appearance (^6^). Limb muscle hypertrophy, especially in the legs, and vein enlargement are remarkable characteristics. Most individuals develop insulin resistance, which frequently evolves into type 2 diabetes (T2D). Other hormonal and metabolic abnormalities include menstrual disorders, moderate to severe hypertriglyceridemia, NAFLD, and hypoalphalipoproteinemia (^3^-^6^).

In patients with atypical FPLD2, particularly those with the exon 11 R582H variant, only laminin A is affected, since laminin C is the product of the alternative splicing encompassing exons 1 to 10 (^7^,^8^). These patients have a milder form of the condition, with less severe fat loss and metabolic symptoms. They may experience mild hypertriglyceridemia and absence of acanthosis nigricans or hirsutism (^5^-^7^,^9^). In this study, we present the case of a female patient with atypical FPLD2, due to a missense variant in exon 11 of the gene encoding Laminin A (rs57830985,c.1745G>A, p.Arg582His).

## CASE REPORT

A 32-year-old woman, presented with a history of oligomenorrhea, hirsutism, increased adipose tissue on the face, neck, and abdomen, as well as marked muscular hypertrophy of the lower limbs since puberty. She was initially diagnosed with polycystic ovary syndrome (PCOS) for which she was treated with combined oral contraceptives, with no clinical improvement. Given her physical appearance - including moon facies and apparent central obesity - we speculated Cushing syndrome diagnosis, which was unequivocally ruled out twice by a normal 24-hour urinary free cortisol and a low-dose dexamethasone suppression test. For the past four years her menstrual abnormalities and hirsutism had significantly worsened and she had been recently diagnosed with type 2 diabetes.

**Figure 1:**
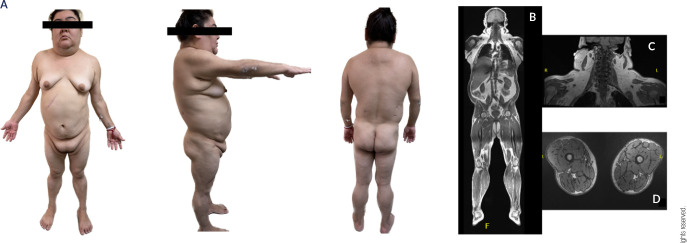
**(A)** Frontal, lateral and posterior view of the patient. Abnormal fat distribution is evident with accumulation of fat in the neck, face, dorsocervical region, and labia majora. Also, a lack of fat in the lower limbs; significant hypertrophy of thighs and calves. **(B, C)** Increased accumulation of subcutaneous adipose tissue in the face, neck, superior chest and back. Hepatomegaly. **(D)** Decreased accumulation of subcutaneous adipose tissue in the lower limbs.

On the physical examination the following measurements were taken: blood pressure of 130/80 mm Hg; pulse 86 beats/min, weight 93 kg, 1.65 m of height, body mass index of 34 kg/m^2^, and 108 cm of waist circumference. The patients presented a striking cushingoid appearance, round, and plethoric face. Severe acanthosis nigricans in the posterior neck, and hirsutism (Ferriman- Gallwey Score 24), as well as a bizarre and abnormal adipose tissue distribution, with fat accumulation in the neck, face, dorsocervical region, and labia majora, accompanied by a total absence of adipose tissue in the lower limbs, and significant hypertrophy of thighs and calves **(Figure 1A)**.

Laboratory evaluation **(Table 1)** revealed severe hypertriglyceridemia, moderate hypercholesterolemia, and uncontrolled diabetes (glycated hemoglobin 9.1%), with a Homeostatic Model Assessment for Insulin Resistance (HOMA-IR) of 23. Prolactin levels, total testosterone, androstenedione, and dehydroepiandrosterone sulfate (DHEA-S) were within normal ranges. Additionally, an adrenocorticotropic hormone (ACTH) stimulation test was performed to rule out nonclassical congenital adrenal hyperplasia, with negative results. Therefore, the diagnosis of polycystic ovary syndrome was concluded **(Table 1)**. A total body MRI confirmed abnormal fat distribution and revealed the presence of a lipoma adjacent to the right trapezius muscle, as well as mild hepatomegaly with grade 1 steatosis **(Figure 1B-D)**. Echocardiography ruled out cardiac structural abnormalities. She was started on high-dose statins and fibrates as well as metformin, sitagliptin, dapagliflozin, insulin glargine, and oral contraceptives.

**Table 1:** Biochemical evaluation

Parameter	Patient values	Reference values
UFC µg/24 h (nmol/d)	49.5 (136.6)	4.3-176 (11.8-485)
LDDST cortisol µg /dL (nnom/L)	0.19 (5.24)	<1.8 (<49.68)
Fasting glucose mg/dL (mmol/L)	212 (11.76)	70-100 (3.9-5.5)
Hb1Ac % (mmol/mol)	9.1 (^45^)	4.0%-5.6% (^20^-^38^)
HOMA-IR	23	<2.5
Triglycerides mg/dL (mmol/L)	1,004 (11.37)	<150 (<1.70)
Total cholesterol mg/dL (mmol/L)	262 (6.78)	<200 (<5.18)
HDL-C mg/dL (mmol/L)	37 (0.95)	>50 (1.29)
Total bilirubin mg/dL (mmol/L)	0.37 (6.29)	0.3-1.20 (5.1-20.5)
AST µ/L (mkat/L)	23 (0.37)	20-48 (0.33-0.80)
ALT µ/L (mkat/L)	34 (0.578)	10-40 (0.17-0.67)
GGT µ/L (mkat/L)	52 (0.78)	9-56 (0.135-0.84)
Alkaline phosphatase U/L (µkat/L)	108 (1.8)	40-150 (0.67-2.5)
FSH mUI/mL (IU/L)	5.01 (5.0)	2.5-12.5 (2.0-12)
LH mUI/mL (IU/L)	7.81 (7.0)	2.4-12.6 (^2^-^18^)
Estradiol pg/mL (pmol/L)	34.12 (122)	19.0-144.0 (68.4-518)
Prolactin ng/mL (nmol/L)	9.39 (0.33)	4.79-23.3 (0.17-1.00)
Total testosterone ng/dL (nmol/L)	32.7 (1.2)	2.0-45 (0.075-1.68)
Androstenedione ng/mL (nmol/L)	1.99 (6.9)	0.3-3.5 (1.04-12.2)
DHEA-S ug/dL (mmol/L)	244.4 (6.6)	45-270 (1.21-7.3)
ACTH Stimulation Test 17α-OH progesterone basal ng/mL (nmol/L) 17α-OH progesterone 60 min ng/mL (nmol/L)	0.9 (2.72) 3.47 (10.5)	<2.0 (6.05) < 5.0 (15.13)

## MATERIALS AND METHODS

### DNA purification

Total DNA was extracted from peripheral blood mononuclear cells. After lysis with proteinase K solution, 300 µL of 5M ammonium acetate were added to precipitate proteins and cellular components. The aqueous phase was transferred to a fresh tube, 600 µL of isopropanol was added and the mixture was incubated overnight at -20 °C, and then centrifuged at 14,000 rpm for 30 min. The resulting DNA pellet was washed with 1 mL 75% ethanol and centrifuged at 10,000 rpm for 5 min; the pellet was air-dried, and DNA resuspended in nuclease free water (^10^).

### Whole-exome sequencing (WES)

The genomic DNA (gDNA) was shipped to the Genomics Core Lab of the *Instituto Tecnológico y de Estudios Superiores de Monterrey* for exome sequencing. Then, gDNA was quantified using Qubit dsDNA BR Assay Kit (Invitrogen, Carlsbad, CA, USA). Quality was determined spectrophotometrically using a Nanodrop One spectrophotometer (Thermo Fisher Scientific, Waltham MA, USA). WES libraries were prepared using Illumina DNA Prep with Exome 1.0 Enrichment (Illumina, San Diego CA, United States). All libraries were quantified with the Qubit dsDNA BR Assay Kit (Invitrogen, Carlsbad, CA, USA), libraries sizes were analyzed in S2 Standard DNA Cartridge for Sep 400 (BiOptic, New Taipei City, Taiwan), and sequencing was performed in a NovaSeq 6000 sequencer (Illumina, San Diego CA, United States) in a 150 bp pair-end configuration.

### FastQC and preprocessing

The quality assessment of the exome libraries was performed with FastQC (Babraham Bioinformatics) to determine the quality of the sequencing. All raw sequences passed the initial quality filter. Adapters were removed and a quality and length filter were performed with Trimmomatic 0.40 (^11^).

### Computational WES analysis

Preprocessed sequences were aligned to the human reference sequence (GRCh38) using the Illumina-Dragen Enrichment pipeline (llumina, San Diego CA, United States). This pipeline was set to produce copy number variants (-enable-cnv true). The BAM files resulting from the enrichment were removed from PCR duplicates using Picard tools (http://broadinstitute.github.io/picard). Each BAM file was used to obtain somatic variants with the GATK pipeline (https://github.com/broadinstitute/gatk/ releases), and variants were annotated by ANNOVAR using the following databases: Clinvar, gnomAD, refGene, cytoBand, exac03, avsnp147, dbnsfp30a (^12^). Genomics were uploaded into the cloud using Docker, GATK, and WDL in Terra. O’Reilly Media. Somatic variants were then transformed to Maf using Funkotator from GATK. Additionally, converted annotated variant files were analyzed with the Maftools package from R programming language to visualize the landscape of critical pathogenic variants.

The pathogenic variants analysis was carried out using Maftools, the variations were filtered using subsetMaf with the parameter “Variant_Classification == Missense_Mutations”. Graphs of pathogenic variants in genes of interest were constructed using lollipop plot to observe variants in general (^13^).

## WES RESULTS

**Figure 2:**
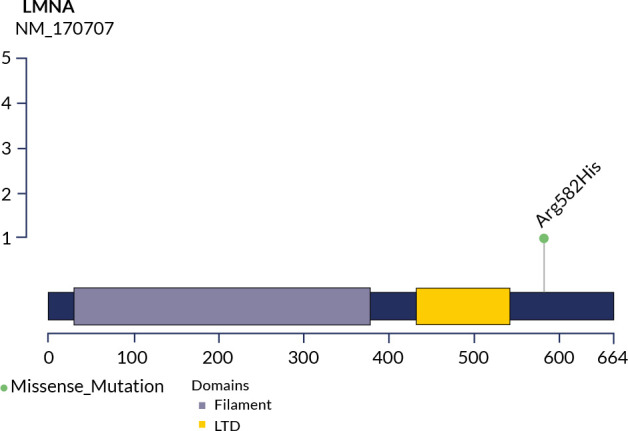
Pathogenic variant in the LMNA gene (rs57830985 c. G1745A, p. R582H)

The analysis identified a heterozygous missense variant of the *LMNA* gene (rs57830985 c. G1745A, p. R582H), a pathogenic variant associated with familial partial lipodystrophy type 2 **(Figure 2)**.

In addition, we searched the whole exome for gene variants linked to glucose metabolism, insulin resistance, adipogenesis, lipid metabolism, and steroidogenesis. **Table 2** presents the aforementioned findings.

**Table 2:** Whole-exome sequencing Results

Gene	Variant	ACMG Classification	Clinical associations
*LMNA*	Type: SNV Genomic position:1:156138534(GRCh38) cDNA: c.1745G>A Protein: p.Arg582His Zygosity: Heterozygous rs57830985	Conflicting classifications of pathogenicity Pathogenic (^2^); Likely pathogenic (^1^); Uncertain significance (^2^)	Familial partial lipodystrophy type 2
*VDR*	Type: SNV Genomic position: 12:47879112 (GRCh38) cDNA: c.2T>C Protein: p.Met1Thr Zygosity: Heterozygous rs2228570	Benign	Inconsistent association with obesity traits. Type 2 diabetes
*PIK3R1*	Type: SNV Genomic position: 5:68292320 (GRCh38) cDNA: c.978G>A Protein: p.Met326Ile Zygosity: Heterozygous rs3730089	Benign/Likely benign	Increased risk of Type 2 diabetes, obesity and dyslipidemia
*GCKR*	Type: SNV Genomic position: 2:27508073 (GRCh38) cDNA: c.1337T>C Protein: p.Leu446Pro Zygosity: Heterozygous rs1260326	Benign	Severe hypertriglyceridemia, non-alcoholic steatohepatitis
*LHCGR*	Type: SNV Genomic position: 2:48694236 (GRCh38) cDNA: c.935A > G Protein: p. Asn312Ser Zygosity: Heterozygous rs2293275	Benign	Acne, hirsutism, high levels of LH and a greater degree of hyperandrogenism, PCOS.
*AKR1C3*	Type: SNV Genomic position: 10:5094459 (GRCh38) cDNA: c.15C>G Protein: p.His5Gln Zygosity: Heterozygous rs12529	Benign	Increased levels of testosterone. Association of insulin resistance with hyperandrogenism.
*CYP21A2*	Type: SNV Genomic position: 6:32040110 (GRCh38) cDNA: c.844G>T Protein: p.Val282Leu Zygosity: Heterozygous rs6471	Pathogenic/Likely pathogenic	Nonclassic congenital adrenal hyperplasia

## DISCUSSION

FPLD2 patients with atypical (and generally heterozygous) pathogenic variants in the *LMNA* gene encoding have different phenotypes and metabolic manifestations than those harboring more typical pathogenic variants (p.Arg482Trp; p.Arg482Gln; p.Arg482Leu; p.Lys486Asn; p.Lys486Asn) (^5^). Garg and cols. reported three patients with the atypical p.R582H pathogenic LMNA variant. These patients had lower levels of triglycerides and a milder limb fat loss than the patients with the classical R482 and K486 pathogenic variants (^5^). Furthermore, heterozygous patients for the atypical p.R582H pathogenic variant have a less severe phenotype and clinical course than homozygous patients, who behave clinically like subjects with typical pathogenic variants (^14^). Neither acanthosis nigricans nor hirsutism are present in patients harboring atypical *LMNA* pathogenic variants, particularly when heterozygous (^5^,^7^). Although our patient was found to have one of these atypical heterozygous *LMNA* pathogenic variants, she displayed a rather severe and aggressive phenotype characterized by a younger age at diagnosis, higher BMI, severe hypertriglyceridemia, marked insulin resistance, hirsutism, and menstrual abnormalities **(Table 3)**. Having sequenced her whole exome, we looked for the concomitant occurrence of pathological variants in genes associated with glucose metabolism, insulin resistance, lipid metabolism, and steroidogenesis that could be associated with the clinical and biochemical characteristics of our patient. In doing so, we found polymorphisms in several genes that participate in different metabolic pathways, including the vitamin D receptor (VDR), the regulatory subunit of phosphaytidylinositol 3-kinase (PIK3R1), the glucokinase regulatory protein (GCKR), the 17β-hydroxysteroid dehydrogenase type 5 (HSD17B5), the LH and chorionic gonadotropin hormone receptor (LHCGR), and the 21-hydroxilase enzyme, among others.

**Table 3:** Comparison of clinical and biochemical characteristics in typical FPLD2, atypical FPLD2 and our patient

Clinical characteristic	Typical FPLD2	Atypical FPLD2	Our patient
Symptoms onset	After puberty	After puberty	After puberty
Age of diagnosis	43 years	42 years	32 years
BMI (kg/m^2^)	24.1	25.4	34
Glucose (mg/dL)	100	88	212
Insulin (µIU/mL)	10.5	6.9	44.26
			
Hb1Ac%	5.6	5.3	9.1
Cholesterol (mg/dL)	216	222	262
HDL-C (mg/dL)	32	42	37
Triglycerides (mg/dL)	573	192	1004
Fat distribution	Fat accumulation in face, neck, armpits, interscapular area, labia majora and visceral. Fat loss in the limbs, buttocks, trunk.	Fat accumulation in face, neck, armpits, interscapular area, labia majora and visceral. Less fat loss in the limbs and buttocks.	Fat accumulation in face, neck, armpits, interscapular area, labia majora and visceral. Fat loss in the limbs and buttocks.
Hirsutism	+	-	+
Acanthosis nigricans	+	-	+
Oligomenorrhea	+	-	+
Calf hypertrophy	++	+	+
Myalgias/muscular weakness	+	-	+
Subcutaneous lipoma	+	-	++
Hepatic steatosis	+	-	+

References: (^5^,^7^,^9^).

The VDR is ubiquitously expressed in various tissues, including muscle, bone, visceral and subcutaneous adipose tissue, liver, and pancreas, and thus participates in several biological processes beyond calcium metabolism (^15^). Our patient harbored the *VDR* gene missense rs2228570 (c.2T>C, p.M1) variant, which modifies the translation initiation codon and results in a shorter protein product that could potentially have a higher binding affinity for 1,25-dihydroxyvitamin D_3_ (^16^,^17^). Although its clinical significance remains controversial (^18^), patients harboring this *VDR* gene variant have been reported to have an increased BMI, waist circumference, total body fat, and sum of skinfold thickness (^15^-^17^, ^19^-^21^). The association of the rs2228570 variant with the development of T2D has been confirmed by several meta-analyses, particularly in Asian populations (^22^-^24^). Even when the exact molecular mechanism is unknown, there is evidence that the VDR regulates insulin synthesis and secretion, and it may have a role in beta cell growth and differentiation, and a protective effect against cytokine mediated inflammation, which contributes to beta cell dysfunction and insulin resistance (^25^,^26^).

The *PIK3R1* gene encodes for the p85 regulatory subunit of the enzyme phosphaytidylinositol 3-kinase (PIK3). This subunit stabilizes the p110 catalytic subunit of the enzyme and determines its biological activity (^27^). WES of our patient revealed the variant rs3730089 (c.978G>A, p.Met326Ile) of the *PIK3R1* gene. This variant leads to a constitutive activation of PI3K, as well as to downstream alterations in protein kinase B (AKT), a signaling cascade that regulates growth, differentiation, survival, and cellular glucose uptake. This variant has been associated with an increased risk of type 2 diabetes, as well as obesity and dyslipidemia (^27^-^29^).

GCKR is an allosteric regulator that modulates the activity and mobilization of glucokinase, favoring a rapid response to changes in glucose concentrations. Our patient had the *GCKR* gene variant rs1260326 (c.1337T>C, p.Leu446Pro), which generates a functional alternative of the protein that promotes glucose uptake and a consequent increment in VLDL and triglyceride synthesis (^30^-^32^). The same variant has been associated with non-alcoholic fatty liver disease and steatohepatitis in Danish, Swedish, Japanese, and Han Chinese populations (^33^-^36^). This variant could contribute to the severe hypertriglyceridemia presented by our patient, in contrast with the mild-moderate hypertriglyceridemia reported in other atypical cases of FPLD2 (^4^-^7^).

The LHCGR is expressed in the ovary (theca and granulosa cells, stromal cells, and luteinized cells) as well as in adipose tissue (^37^,^38^). Its function is essential for sexual differentiation in fetuses and reproductive function in adult life (^38^,^39^). The LHCGR polymorphism (rs2293275, c.935A > G, p. Asn312Ser) found in our patient is a missense single-nucleotide variant, identified as a risk factor for the development of PCOS (^39^-^41^), although the evidence is controversial (^42^). This variant is associated with an abnormal glycosylation of the LHCGR, which may affect its trafficking, stability, and sensitivity (^43^). These alterations have been associated with abnormal LH signaling, which seems to have a key role in ovarian androgen production, follicular development, and ovulation (^43^,^44^).

The patient also has a variant in the HSD17B5 gene. This enzyme is a member of the aldo-keto reductase (AKR) superfamily. It catalyzes the synthesis of potent androgens in peripheral tissues (theca cells and adrenal glands), converting androstenedione to testosterone, activating the androgen receptor, and acting as a substrate for aromatase (^45^,^46^). The rs12529 (c.15C>G, p.His5Gln) is a non-synonymous single nucleotide variant in exon 1 of the *HSD17B5* gene that involves a buried charge change and thus results in a more unstable protein product (^47^,^48^). The clinical impact of rs12529 in women is controversial. Some studies report its association with increased levels of testosterone and insulin resistance (^49^,^50^).

Finally, our patient also harbored a variant of the *CYP21A2* gene (rs6471, c.844G>T, p.Val282Leu), which encodes the steroidogenic enzyme 21-hydroxilase. This polymorphism reduces the enzymatic activity by 50%-80% and is associated with non-classic congenital adrenal hyperplasia (^51^-^53^). The heterozygous condition of this variant implies that a single normal allele in the patient was enough to preclude the appearance of the associated phenotype as reported by Inácio and cols. (^54^). Adrenal hyperplasia was excluded with an ACTH stimulation test during evaluation for hirsutism and amenorrhea.

Our case suggests that the atypical manifestations of FPLD2 are associated with pathogenic variants in the laminin genes, but they also may be related to variations in other genes. Several genes are required to regulate different metabolic pathways. The presence of other polymorphisms and pathogenic variants may change the phenotype of atypical cases. Other case studies could help clarify if the alterations detected in our patient are common in cases with aggressive metabolic manifestations. This case is an example of the differential diagnostic algorithm in patients with severe metabolic conditions in which other neuroendocrine causes have been suspected and excluded.

An important limitation of this study is that the exome analysis was not performed in relatives of the patient. An evaluation of family members with and without manifestations of metabolic disease and lipodystrophy would be ideal for calculating the weight of the exome variations in the phenotype of the patient.

It is important to understand the benefits and limitations of exome sequencing in order to interpret the clinical relevance of genomic variants. In our case, we tried to identify clinical and biochemical aspects that did not coincide with what has been reported in the literature, and we looked for genetic variants that could be associated with these characteristics. The clinical significance of the variants found in our patient is still subject to debate, since their association with metabolic alterations has not been consistent in all studies.

In conclusion, the clinical and biochemical characteristics of patients with heterozygous FPLD2 harboring an atypical variant (rs57830985, c.1745G>A, p.Arg582His) are rare. Exome sequencing can contribute to the understanding of unusual or aggressive phenotypes. The true contribution of other genetic variants detected in the exomes of such patients requires validation in larger cohorts of patients and genetically similar populations.

## Data Availability

whole-exome sequencing data is available by contacting Etual Espinosa-Cardenas (espinosaetual@gmail.com) upon reasonable request.
